# Ginseng metabolite Protopanaxadiol induces Sestrin2 expression and AMPK activation through GCN2 and PERK

**DOI:** 10.1038/s41419-019-1548-7

**Published:** 2019-04-05

**Authors:** Hong Ri Jin, Charles H Du, Chong-Zhi Wang, Chun-Su Yuan, Wei Du

**Affiliations:** 1 Ben May Department for Cancer Research, University of Chicago, Chicago, IL 60637 USA; 2 Pritzker School of Medicine, University of Chicago, Chicago, IL 60637 USA; 3 Tang Center for Herbal Medicine Research, University of Chicago, Chicago, IL 60637 USA; 4 Department of Anesthesia and Critical Care, University of Chicago, Chicago, IL 60637 USA; 5 Present Address: Department of Pathology and Laboratory Medicine, Indiana University School of Medicine, Indianapolis, IN 46202 USA

## Abstract

Ginseng is one of the most commonly used herbs that is believed to have a variety of biological activities, including reducing blood sugar and cholesterol levels, anti-cancer, and anti-diabetes activities. However, little is known about the molecular mechanisms involved. In this study, we showed that protopanaxadiol (PPD), a metabolite of the protopanaxadiol group ginsenosides that are the major pharmacological constituents of ginsengs, significantly altered the expression of genes involved in metabolism, elevated Sestrin2 (Sesn2) expression, activated AMPK, and induced autophagy. Using CRISPR/CAS9-mediated gene editing and shRNA-mediated gene silencing, we demonstrated that Sesn2 is required for PPD-induced AMPK activation and autophagy. Interestingly, we showed that PPD-induced Sesn2 expression is mediated redundantly by the GCN2/ATF4 amino acid-sensing pathway and the PERK/ATF4 endoplasmic reticulum (ER) stress pathway. Our results suggest that ginseng metabolite PPD modulates the metabolism of amino acids and lipids, leading to the activation of the stress-sensing kinases GCN2 and PERK to induce Sesn2 expression, which promotes AMPK activation, autophagy, and metabolic health.

## Introduction

Autophagy is an evolutionarily conserved self-digestive process via which cells adapt to nutrient starvation and other stress conditions^1,2^. Defects in autophagy is associated with a number of pathological conditions, including cancer, infectious diseases, myopathies, and neurodegenerative disorders^3,4^. Under normal growth conditions, autophagy is kept at a basal level mainly for housekeeping purposes such as degradation of long-lived proteins and turnover of damaged cellular organelles. Under stress conditions such as nutrient starvation, ER stress, oxidative stress, and hypoxia, autophagy is induced to provide cells with additional internal nutrient supplies and promote cellular survival^5–8^. The molecular pathway that links nutrient depletion and stress to autophagy involves the energy sensor AMP-activated protein kinase (AMPK) and the mechanistic target of rapamycin complex 1 (mTORC1). AMPK was shown to be associated with ULK1 (unc-51-like kinase 1) and phosphorylation of ULK1 by AMPK was required for autophagy initiation^9^. In addition, AMPK can also promote autophagy by inhibition of autophagy-negative regulator mTORC1^10^.

Sestrin2 (Sesn2) is a member of the highly conserved antioxidant proteins, which is induced by a variety of cellular stress and functions to activate AMPK, inhibit mTORC1, and suppress reactive oxygen species accumulation^11–14^. This allows cells to adjust their metabolism to adapt to different cellular stresses. Consistent with this, in vivo studies revealed that Sesn2 is important for metabolic homeostasis. Sesn2-deficient mutants showed diverse age- and obesity-associated metabolic pathologies such as accumulation of lipid droplets and protein aggregates, mitochondrial dysfunction, and insulin resistance. These pathologies were related to defective autophagy and the misregulation of AMPK and mTORC1 signaling^15–17^. Importantly, restoration of AMPK activation suppressed the observed defects in Sestrin-deficient fly and mouse mutants^16–18^, suggesting activation of AMPK is a key in vivo function of Sesn2. In addition, Sesn2 can serve as a Leu sensor and regulate mTORC1 activity directly through modulation of GATOR complexes^19^.

In response to different environmental stresses, the α-subunit of eukaryotic initiation factor 2 (eIF2α) is phosphorylated by distinct kinases, which represses global translation initiation but selectively enhances the translation of ATF4, a master regulator controlling the transcription of downstream target genes essential for adaptive responses, including genes involved in metabolism, cell survival, apoptosis, and autophagy^20–22^. In mammals, four protein kinases, GCN2, PERK, HRI, and PKR, are known to phosphorylate eIF2α in response to distinct upstream stress signals to modulate translation initiation. The GCN2 kinase, which is activated by binding to the uncharged tRNA, phosphorylates eIF2α in response to amino acid deprivation, whereas PERK, which is a transmembrane protein of the ER, phosphorylates eIF2α in response to ER stress^21,23^.

Ginseng is one of the most commonly used herbs with a variety of biological activities. Evaluation of randomized controlled trials revealed that *Panax ginseng* showed promising results for improving glucose metabolism, reducing inflammation, and preventing cancer recurrence^24^. However, little is known of how ginseng may exert its various effects. It is generally accepted that the major pharmacological constituents of ginsengs are ginsenosides with the majority being the protopanaxadiol (PPD) group ginsenosides^25^. After oral ingestion, the bioavailability of ginseng parent compounds is low due to the low level of absorption. Instead, the parent compounds are transformed by the intestinal microbiome to the more easily adsorbed metabolites such as compound K and PPD^26–28^. We showed previously that PPD significantly affected expression of genes involved in lipid and steroid biosynthesis, and induced ER stress, pro-death mechanisms such as p53, and pro-survival mechanisms such as autophagy^29^. Recent studies suggest that defective autophagy, which is regulated by AMPK and mTOR, is linked with metabolic diseases such as obesity, diabetes, and its complications^30^. In this study, we characterized the mechanisms by which PPD induces autophagy.

## Materials and methods

### PPD source and quality

PPD was synthesized and purified as described^26^. The high-performance liquid chromatography-determined purity was 95.3%.

### Cell culture, chemicals, and reagents

Human colorectal cancer cells HCT116 were obtained from the American Type Culture Collection. Cells were maintained in Dulbecco’s modified Eagle’s medium supplemented with 5% fetal bovine serum (Hyclone Laboratories), 50 IU of penicillin/streptomycin (Gemini Bio-Products), and 2 mmol/l of l-glutamine (Invitrogen) in a humidified atmosphere with 5% CO_2_ at 37 °C. The p53^−/−^ HCT116 cells were described previously^31,32^. PERK^+/+^ and PERK^−/−^ mouse embryonic fibroblasts (MEFs) were described previously^33,34^. NAC (*N*-acetylcysteine) was obtained from Sigma.

### Western blot analysis

After being treated for the desired period of time, HCT116 cells were collected and washed twice with phosphate-buffered saline and lysed in RIPA buffer (20 mM Tris-HCl, 150 mM NaCl, 1 mM EDTA, 1 mM EGTA, 1% NP-40, 1% sodium deoxycholate, 1 mM phenylmethlsulfonyl fluoride, 2.5 mM sodium pyrophosphate, 1 mM β-glycerophosphate, 1 mM sodium vanadate, 1 µg/ml leupeptin). Equal amounts of protein were loaded and the immunoblotting was detected by Li-Cor Odyssey image reader as described^32^. Anti-β-actin antibody was obtained from Santa Cruz Biotechnology. Antibodies against LC3-I/II, phospho-AMPKα (Thr172), ACC, phospho-ACC (Ser79), and PERK were obtained from Cell Signaling Technology. Anti-Sesn2 antibody was obtained from Proteintech.

### TFEB-GFP nuclear localization analysis

Wild-type (WT) or Sesn2 knockout cells were transfected with TFEB-GFP plasmid (a gift from Shawn Ferguson, Addgene plasmid #38119). Forty-eight hours after transfection, cells were treated with vehicle control or 30 µM PPD for 20 h and the localization of TFEB-GFP were analyzed by fluorescence microscopy. To determine the percentage of cells with nuclear TFEB-GFP, the localization of randomly selected 20 cells for each treatment groups were determined and repeated 3 times.

### RNA isolation and RT-PCR

Total RNA was extracted with the RNeasy Mini Kit according to the manufacturer’s instructions (Qiagen). cDNA was synthesized using M-MLV reverse transcriptase from Promega. Quantitative real-time PCR was carried out as described^35^. Primer pairs used for reverse-transcription PCR (RT-PCR) are: human Sesn2, 5′-CTCCTG GCGCCACTACATTG-3′ (sense) and 5′-ACTCAGGGTCACCACCAGT-3′ (antisense); human CHOP, 5′-TGGAAGCCTGGTATGAGGAC-3′ (sense) and 5′-TGTGACCTCTGCTGGTTCTG-3′ (antisense); human ATF3, 5′-GCAGAGCTAAGCAGT CGTGG -3′ (sense) and 5′-CGCCT TGATGGTTCTCTGCT-3′ (antisense); human GAPDH, 5′-CTCTGACTTCAACAGCGACAC-3′ (sense) and 5′-CATACCAGGAAATGAGCTTGACAA-3′ (antisense); mouse Sesn2, 5′-GCACCTTCGCCTCCCAGTGA-3′ (sense) and 5′-GCAGGCTCTCCTGAAGCTGC-3′ (antisense); mouse GAPDH, 5′-GCACAGTCAAGGCCGAGAAT-3′ (sense) and 5′-GCCTTCTCCATGGTGGTGAA-3′ (antisense).

### Microarray data analysis

The data from six microarrays of control or PPD-treated samples were obtained and normalized as described previously^36^. The normalized data were analyzed using ArrayTools Version 4.3, to identify genes significantly affected by 20–25 µM of PPD treatment. Replicates were averaged by geometric mean. Parametric *p*-values were computed using *t*-tests on the logarithms of gene expression intensities. Genes with significantly altered expression levels, grouped by Kegg biochemical pathway annotations and ordered by the smallest *p*-value within the pathway group, are shown in Supplementary Table S1. Significance Analysis of Microarrays was performed with a delta of 0.8 and target false discovery rate of 0.001^37^. Genes were excluded if <20% of expression data exhibited a 1.5-fold change from the gene’s median or >50% of data were missing. A minimum intensity threshold was set at 10. Genes identified by the analysis are listed in Supplementary Table S2.

### Lentiviral preparation and transduction

The pLKO.1 lentiviral sh-RNA expression system was used to generate short hairpin RNA (shRNA) constructs as described previously^38^. The sequences of shRNA and SgRNA used in this study included the following: sh-GFP (5′-ACGTCTATATCATGGCCGACA-3′), sh-Sesn2 (5′-GGTCCACGTGAACTTGCT GC-3′). Lentiviral packaging was done according to the previously described protocol^39^. Briefly, expression plasmids pCMV-dR8.91 and pCMV-VSV-G were co-transfected into 293T cells using the calcium phosphate method at 20:10:10 μg (for a 10 cm dish). The transfection medium containing calcium phosphate and plasmid mixture was replaced with fresh complete medium after incubation for 5 h. Media containing virus was collected 48 h after transfection and then concentrated using 20% sucrose buffer at 20,000 × *g* for 4 h. The virus pellet was re-dissolved in the proper amount of complete growth medium and stocked at −80 °C. Cells were infected with the viruses at the titer of 100% infection in the presence of Polybrene (10 μg/ml) for 48 h and were treated as desired.

The Lenti-Crispr V2 system was used to generate knockout clones^40^: Sg-GCN2 (5′-GGAGAGCTACCCGCAACGAC-3′), Sg-PERK (5′-TGGAGCGCGCCATCAGCCCG-3′), Sg-ATF4 (5′-AGTGAAGTGGATATCACTGA-3′), Sg-Sesn2 (5′-GGACTACCTGCGGTTCGCCC-3′), Sg-AMPKα1 (5′-ATTCGGAGCCTTGATGTGGT-3′), and Sg-AMPKα2 (5′-ATTCGCAGTTTAGATGTTGT-3′). To generate HCT116 cells with single-gene knockout, cells were infected with SgRNA viruses at the titer of 100% infection in the presence of Polybrene (10 μg/ml) for 48 h and then single-cell clones was selected by serial dilution in 96-well plate. To generate GCN2 and PERK double mutant clones (DKO GCN2&PERK), sgRNA against PERK was introduced into a clone of GCN2 single knockout cells and then single-cell clones was selected by serial dilution in 96-well plate. The single-cell clones were confirmed by genomic DNA sequencing or western blotting.

## Results

### PPD induces Sesn2 expression, which is required for PPD-induced autophagy

**Fig. 1:**
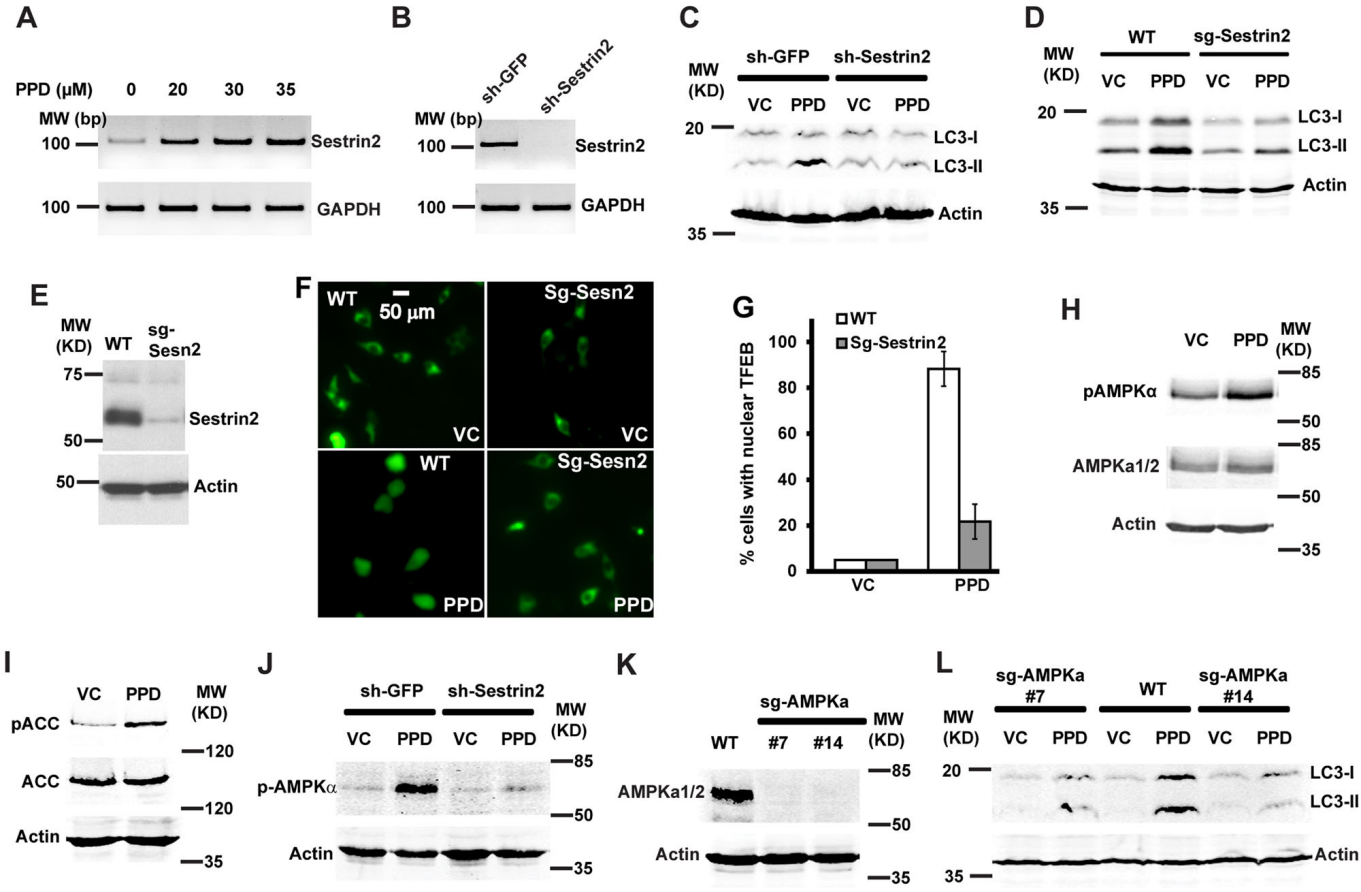
PPD induces Sestrin2, which is required for the induction of AMPK and autophagy. **a** HCT116 cells were treated with different concentrations of PPD for 24 h and then the expression levels of Sestrin2 was determined by RT-PCR. **b** RT-PCR showing knockdown of Sestrin2 expression by sh-Sestrin2 construct. **c** HCT116 cells were infected with sh-Sestrin2 constructs or sh-GFP control. The knockdown cells were treated with 30 µM PPD for 24 h and then the LC3-I/II protein levels were determined by western blottings. **d** HCT116 cells were infected with sg-Sestrin2 or sg-control constructs. The pools of Sestrin2 knockout or Control cells were treated with PPD 30 µM for 24 h and then the LC3-I/II protein levels were determined by western blottings. **e** Western blotting result data showing that sg-Sestrin2-targeted cells has significantly decreased Sestrin2 protein levels. **f** Localization of TFEB-GFP in WT or sg-Sestrin2-targeted cells were analyzed by fluorescence microscopy after treatment with vehicle control (VC) or 30 µM PPD for 20 h. The white scale bar indicate 50 µm. **g** The average percentage of cells with nuclear TFEB-GFP from **f** were quantified. The average and SD for each treatment groups were shown. **h** HCT116 cells were treated with PPD 30 µM for 24 h and then the level of total AMPKα and phosphorylated AMPKα (Thr172) was determined by western blottings. **i** HCT116 cells were treated with PPD 30 µM for 24 h and then the level of phosphorylated ACC (Ser79) was determined by western blottings. **j** HCT116 cells were infected with sh-Sestrin2 or sh-GFP control constructs. The knockdown cells and were treated with PPD 30 µM for 24 h and then the p-AMPKα (Thr172) protein levels were determined by western blottings. **k** Western blotting showing the elimination of the AMPKα protein in Crispr/Cas9-mediated AMPKα knockout cells. **l** HCT116-WT and HCT116-AMPKα-knockout cells were treated with PPD 30 µM for 24 h and then the LC3-I/II protein levels were determined by western blottings

We examined PPD-induced gene expression alterations to gain insights into the mechanisms by which PPD may regulate autophagy. Analysis of microarray expression profiling data for significantly altered expression of individual genes nominated differences in the metabolism of various amino acids, purine and pyrimidine, propanoate and butanoate, Glycerophospholipid, in the biosynthesis of steroids and aminoacyl-tRNA, and in mitogen-activated protein kinase and Insulin signaling (Supplementary Table S1). Significance analysis of microarrays for PPD-altered genes identified Sesn2, an important regulator of metabolic homeostasis, in addition to several genes involved in amino acid transport and synthesis such as SLC7A11, SLC6A9, and Asparagine Synthetase (Supplementary Table S2). Sesn2 has been shown to regulate AMPK and mTOR, which are key regulators of autophagy. Indeed, Sesn2 expression was highly induced by PPD (Fig. 1a). As Sesn2 is known to regulate AMPK and autophagy, and we previously showed that PPD induced autophagy, we determined whether Sesn2 is required for PPD-induced autophagy by examining the effects of Sesn2 inactivation. PPD treatment significantly increased the level of LC3-II (Fig. 1c, d, sh-GFP and WT). TFEB is transcription factor that regulates expression of autophagy-regulated genes and its nuclear localization can be used to evaluate autophagic flux^41^. We used TFEB-GFP cytoplasmic and nuclear localization to further analyze autophagy induction by PPD^42^. TFEB-GFP was excluded from the nucleus in control-treated WT cells; PPD treatment induced TFEB-GFP to be present in the nucleus (Fig. 1f, g, WT), similar to treatment with serum-free medium that is known to induce autophagy (Supplementary Fig. S1). These results suggest that PPD induces autophagy in WT HCT116 cells. Interestingly, inactivation of Sesn2 either by shRNA knockdown (Fig. 1b) or by CRISPR/CAS9-mediated knockout (Fig. 1e) significantly inhibited PPD-induced LC3-II accumulation (Fig. 1c, d) and blocked PPD-induced presence of TFEB-GFP in the nucleus (Fig. 1f, g). These results suggested that PPD-induced Sesn2 is required for its induction of autophagy.

### Sesn2 is required for PPD-induced AMPK activation

Sesn2 is known to regulate AMPK activity^43^. The induction of Sesn2 by PPD suggest that PPD may induce AMPK activation, which can be monitored by phosphorylation on AMPKα T172^44^. Indeed, PPD treatment significantly increased p-AMPKα levels (Fig. 1h) and significantly increased phosphorylation of ACC on Ser79 (Fig. 1i), a key target of AMPK in fatty acid biosynthesis^45^. These results show that PPD induces AMPK activation. Interestingly, PPD also slightly increased levels of AMPKα (Fig. 1h), which is consistent with the report that Sesn2 also modulates AMPK subunit expression^46^. Importantly, knockdown of Sesn2 significantly inhibited PPD-induced phosphorylation of AMPKα on T172 (Fig. 1j). These results indicate that Sesn2 is also required for PPD-induced AMPK activation.

TORC1 and ULK1 kinase complexes are key regulators of autophagy. As AMPK has been shown to regulate both TORC1 and ULK1 kinase complexes, we tested whether AMPK activation is involved in PPD-induced autophagy. We used CRISPR/CAS9 approach to knock out both AMPKα1 and AMPKα2. Double knockout (DKO) AMPKα1/α2, which removed both AMPKα1 and AMPKα2 proteins (Fig. 1k), significantly decreased PPD-induced LC3-II accumulation (Fig. 1l). Taken together, these results suggest that Sesn2 is required for PPD-induced AMPK activation, which contributes to the autophagy induction.

### PPD-induced Sesn2 expression requires ATF4

**Fig. 2:**
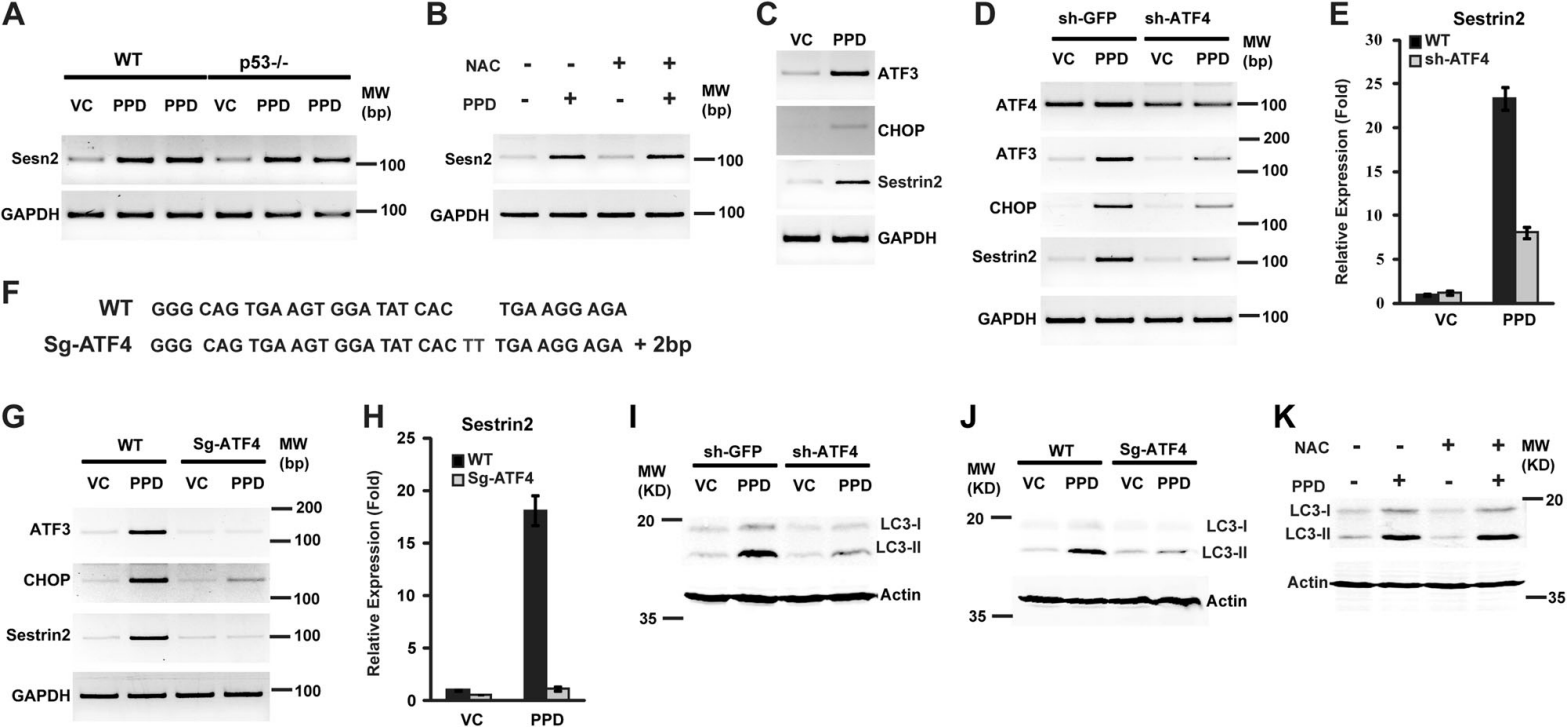
ATF4 is required for PPD-induced Sestrin2 expression and autophagy. **a** HCT116 (P53^+/+^) and HCT116 (P53^−/−^) cells were treated with PPD 30 µM for 24 h and then the expression level of Sestrin2 was determined by RT-PCR (two PPD treatment group by same concentration). **b** HCT116 cells were treated with PPD 30 µM in the absence or presence of NAC for 24 h and then the expression levels of Sestrin2 was determined by RT-PCR. **c** HCT116 cells were treated with PPD 30 µM for 24 h and then the expression levels of ATF3, CHOP, and Sestrin2 were determined by RT-PCR. **d** HCT116 cells were infected with sh-ATF4 constructs or sh-GFP control. The knockdown cells were treated with PPD 30 µM for 24 h and then the expression level of ATF3, CHOP, and Sestrin2 were determined by RT-PCR. **e** HCT116 cells were infected with sh-ATF4 constructs or sh-GFP control. The knockdown cells were treated with PPD 30 µM for 24 h and then the expression of Sestrin2 was determined by qRT-PCR. **f** Genomic sequencing data showing there are 2 bp insertion in the ATF4 open reading frame. **g** HCT116 and HCT116-ATF4 KO cells were treated with PPD 30 µM for 24 h and then the expression of ATF3, CHOP, and Sestrin2 were determined by RT-PCR. **h** HCT116 and HCT116-ATF4 KO cells were treated with PPD 30 µM for 24 h and then the expression of Sestrin2 was determined by qRT-PCR. **i** HCT116 cells were infected with sh-ATF4 constructs or sh-GFP control. The knockdown cells were treated with PPD 30 µM for 24 h and then the expression level of LC3-I/II were determined by western blotting. **j** HCT116 and HCT116-ATF4 KO cells were treated with PPD 30 µM for 24 h and then the expression level LC3-I/II were determined by western blotting. **k** HCT116 cells were treated with PPD 30 µM in the absence or presence of NAC for 24 h and then the expression level of LC3-I/II were determined by western blotting

Sesn2 has been shown to be induced by different stress insults, including p53, oxidative stress, and ER stress. To determine the mechanism by which PPD induces Sesn2, we examined the effects of inactivating p53, addition of antioxidant NAC, or inactivation of ATF4, a key transcription factor in the PERK branch of ER stress response. Knockout of p53 or addition of NAC did not affect Sesn2 induction by PPD (Fig. 2a, b). These observations suggest that PPD-induced Sesn2 is not mediated by p53 or oxidative stress. In contrast, knockdown of ATF4 significantly decreased PPD-induced Sesn2 expression (Fig. 2d, e). In addition, PPD also induced expression of ATF3 and CHOP (Fig. 2c), two ATF4-regulated transcription factors regulating the expression of genes involved in the stress response program^21^. As expected, induction of ATF3 and CHOP by PPD was also significantly inhibited by ATF4 knockdown (Fig. 2d). In addition, we used CRISPR/CAS9 approach to knock out ATF4 and generated an ATF4 mutant line with a 2 bp deletion in the open reading frame (Fig. 2f). Knockout of ATF4 blocked PPD-induced expression of Sesn2, ATF3, and CHOP (Fig. 2g, h). Furthermore, inactivation of ATF4 but not addition of NAC inhibited PPD-induced LC3-II accumulation (Fig. 2i–k). These results suggest that ATF4 is required for PPD-induced Sesn2 expression, which in turn is required for PPD-induced autophagy.

### Inactivation of PERK alone does not inhibit PPD-induced Sesn2 expression

**Fig. 3:**
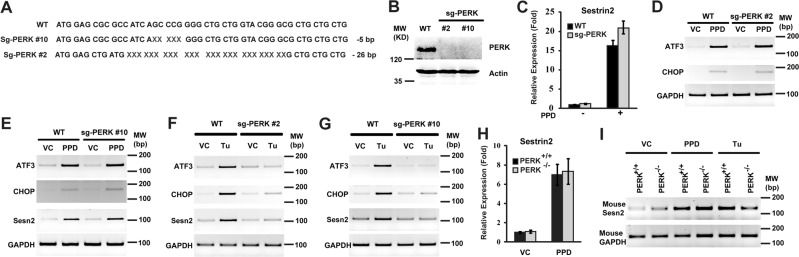
PERK is not necessary for PPD-induced Sestrin2 expression. **a** Genomic sequencing data showing there are 5 bp and 26 bp deletion in the PERK open reading frame in two independent PERK knockout clones. **b** Western blotting showing the elimination of PERK protein in the PERK knockout cells. **c** HCT116-WT and HCT116-PERK KO (clone No. 2) cells were treated with PPD 30 µM for 24 h and then the expression level of Sestrin2 was determined by qRT-PCR. **d** HCT116-WT and HCT116-PERK KO (clone No. 2) were treated with PPD 30 µM for 24 h and then the expression level of ATF3 and CHOP were determined by RT-PCR. **e** HCT116-WT and HCT116-PERK KO (clone No. 10) were treated with PPD 30 µM for 24 h and then the expression level of ATF3, CHOP, and Sestrin2 were determined by RT-PCR. **f** HCT116-WT and HCT116-PERK KO cells (clone No. 2) were treated with ER stress inducer Tunicamycin (10 μg/ml) for 20 h and then the expression levels of ATF3, CHOP, and Sestrin2 were determined by RT-PCR. **g** HCT116-WT and HCT116-PERK KO cells (clone No. 10) were treated with ER stress inducer Tunicamycin (10 µg/ml) for 20 h and then the expression levels of ATF3, CHOP, and Sestrin2 were determined by RT-PCR. **h** PERK^+/+^ and PERK^−/−^ mouse embryonic fibroblasts (MEFs) were treated with PPD 30 µM for 24 h and then the expression level of Sestrin2 was determined by qRT-PCR. **h** PERK^+/+^ and PERK^−/−^ MEFs were treated with PPD 30 µM for 20 h or Tunicamycin (10 µg/ml) for 20 h and then the expression level of Sestrin2 was determined by RT-PCR

As ATF4 is activated by PERK during ER stress and as PPD can induce ER stress and expression of PERK branch of ER stress targets CHOP and ATF3, we further examine the effect of inactivating PERK. Two independent PERK small deletion lines that caused frame-shift mutations were generated using CRISPR/CAS9 approach (Fig. 3a). Both lines eliminated PERK protein (Fig. 3b). However, knockout PERK did not decrease PPD-induced expression of Sesn2, CHOP, or ATF3 (Fig. 3c–e). Tunicamycin (Tu), a well-characterized ER stress-inducing agent, induces ER stress because of the inhibition of *N*-linked glycoslylation and the accumulation of unfolded glycoproteins in the ER. To make sure that the PERK-knockout cell lines did have the expected effects on ER stress, we characterize their effects on Tu-induced ER stress. Tu treatment in WT cells induced expression of Sesn2, CHOP, and ATF3. Knockout of PERK significantly inhibited Tu-induced expression of these genes (Fig. 3f, g). Therefore, the PERK knockout cells failed to inhibit PPD-induced Sesn2 expression, despite having the expected function of blocking Tu-induced expression of PERK branch of ER-stress target genes. Furthermore, PPD-induced similar levels of Sesn2 expression in matched PERK^+/+^ and PERK^−/−^ MEFs, even though Tu-induced Sesn2 was reduced in the PERK^−/−^ cells (Fig. 3h, i). Taken together, these results suggest that PPD may induce ATF4 activation and Sesn2 expression through a pathway in parallel to PERK.

### Both amino acid deprivation and ER stress induce Sesn2 expression through ATF4

**Fig. 4:**
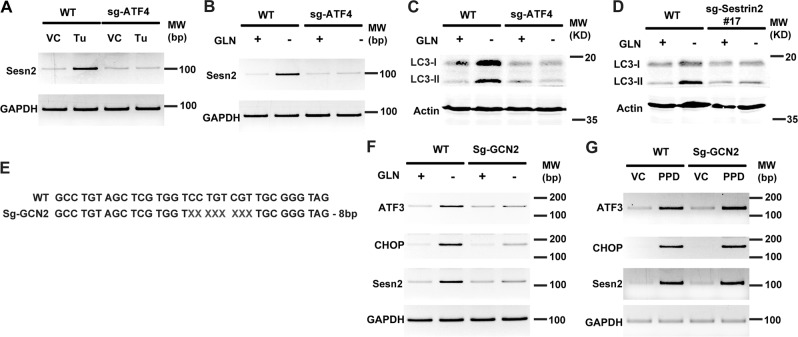
ATF4 is required for ER stress and glutamine deprivation-induced Sestrin2 expression and autophagy. **a** HCT116-WT and HCT116-ATF4 KO cells were treated with tunicamycin (10 µg/ml) for 20 h and then the expression of Sestrin2 was determined by RT-PCR. **b** HCT116-WT and HCT116-ATF4 KO cells were treated with glutamine (GLN) deprivation for 20 h and then the expression of Sestrin2 was determined by RT-PCR. **c** HCT116-WT and HCT116-ATF4 KO cells were treated with glutamine (GLN) deprivation for 20 h and then the level of LC3-I/II were determined by western blotting. **d** HCT116-WT and HCT116-Sestrin2 KO cells were treated with glutamine (GLN) deprivation for 20 h and then the level of LC3-I/II were determined by western blotting. **e** Genomic sequencing data showing there are 8 bp deletion in the GCN2 open reading frame. **f** HCT116-WT and HCT116-GCN2 KO cells were treated with glutamine (GLN) deprivation for 20 h and then the expression of ATF3, CHOP, and Sestrin2 were determined by RT-PCR. **g** HCT116-WT and HCT116-GCN2 KO cells were treated with PPD 30 µM for 24 h and then the expression of ATF3, CHOP, and Sestrin2 were determined by RT-PCR

ATF4 functions downstream of PERK to activate this branch of ER-stress-induced target gene expression. Indeed, ATF4 knockout blocked Tu-induced Sesn2 expression (Fig. 4a). As PPD-induced Sesn2 expression was inhibited by inactivation of ATF4 but not PERK, we tested the possibility that PPD may induce another pathway to activate ATF4. Amino acid deprivation is known to activate ATF4^21^. Indeed, deprivation of amino acid Gln induced Sesn2 expression, which was blocked by ATF4 knockout (Fig. 4b). Furthermore, LC3-II accumulation induced by Gln deprivation was also inhibited by inactivation of ATF4 or Sesn2 (Fig. 4c, d). These results show that ATF4 is required for amino acid deprivation and ER stress-induced Sesn2 expression as well.

### GCN2 and PERK redundantly regulate PPD-induced Sesn2 expression

As GCN2 can upregulate ATF4 in response to amino acid deprivation, we generated GCN2-knockout cells with CRIPSR/Cas9 approach to characterize the effect of inactivating GCN2 on PPD-induced Sesn2 expression (Fig. 4e). Interestingly, inactivation of GCN2 inhibited Gln-deprivation-induced Sesn2, CHOP, or ATF3 expression (Fig. 4f) but did not inhibit PPD-induced Sesn2, CHOP, or ATF3 expression (Fig. 4g). These results suggest that inactivation of GCN2 alone is not sufficient to block for PPD-induced Sesn2 expression either.

**Fig. 5:**
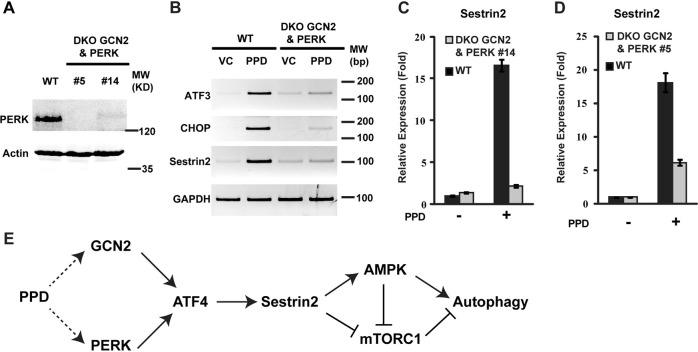
Double knockout of GCN2 and PERK inhibits PPD-induced Sestrin2 expression. **a** Extracts from HCT116-WT and HCT116-GCN2 and PERK double knockout cells (DKO GCN2 and PERK) were analyzed by western blottings to determine the levels of PERK protein. **b** HCT116-WT and HCT116-DKO GCN2 and PERK cells were treated with PPD 30 µM for 20 h and then the expression of ATF3, CHOP, and Sestrin2 were determined by RT-PCR. **c** HCT116-WT and HCT116-DKO GCN2&PERK-No. 14 cells were treated with PPD 30 µM for 20 h and then the expression of Sestrin2 was determined by qRT-PCR. **d** HCT116-WT and HCT116-DKO-GCN2&PERK-No. 5 cells were treated with PPD 30 µM for 20 h and then the expression of Sestrin2 was determined by qRT-PCR. **e** A model summarizing the mechanisms by which PPD induces Sestrin2 expression and autophagy

To test the possibility that PPD activates both GCN2 and PERK redundantly to upregulate ATF4, we generated two independent GCN2 and PERK DKO cell lines from the GCN2 single knockout cells shown in Fig. 4e. As expected, both DKO cells reduced PERK protein (Fig. 5a). Importantly, both DKO cells significantly reduced PPD-induced expression of Sesn2, CHOP, and ATF3 (Fig. 5b–d). These results suggest that PPD can activate ATF4 and Sesn2 expression through either GCN2 or PERK, which functions redundantly downstream of PPD to upregulate ATF4 (Fig. 5e).

## Discussion

Our results showed that Sesn2 is strongly induced by ginseng metabolite PPD and is required for PPD to activate AMPK and induce autophagy (Fig. 5e). Expression of Sesn2 can potentially be induced by a variety of stress signals. Interestingly, although inactivating GCN2 or PERK alone can block amino acid deprivation or ER stress-induced Sesn2 expression, respectively, inactivating either GCN2 or PERK failed to block PPD-induced Sesn2 expression. In contrast, inactivation of both GCN2 and PERK together blocked PPD-induced Sesn2 expression. These results suggest that PPD treatment activate both GCN2 by causing amino acid deprivation and PERK by inducing ER stress (Fig. 5e).

As PPD significantly affected expression of genes involved in amino acid transport, metabolism, and synthesis of aa-tRNA (Table S1), it is likely to be that PPD treatment caused deficiency in the level of certain amino acid and certain aa-tRNA, which induced GCN2 activation. In addition, PPD also significantly inhibited expression of genes involved in fatty acid and cholesterol biosynthesis, and interfered with normal lipid metabolism, which may induce ER stress and PERK activation directly through causing lipid disequilibrium^47,48^. Consistent with this, we found that PPD-induced ER stress was not significantly affected by inhibition of new protein synthesis^29^.

It is increasingly clear that activation of AMPK, induction of autophagy, and inhibition of mTORC1 are associated with increased metabolic health. Metabolic syndrome is a disorder in the balance between energy supply, utilization, and storage, and is linked with obesity, insulin resistance, development of type 2 diabetes, and increased risks of developing cardiovascular disease and many types of cancers^49–51^. Extensive evidence suggest that dysregulation of AMPK is associated with these metabolic disorders, and that modulation of AMPK activation, either through excise or by pharmacological AMPK activators, could be used to treat or prevent these diseases^52^. AMPK activation reprograms cellular metabolism, induces autophagy, and promotes mitochondria homeostasis^53^. Therefore, the observed activation of AMPK by ginseng metabolite PPD raise the possibility that ginseng may have beneficial effects to promote metabolic health. This is consistent with finding from a systematic review of randomized controlled trials that showed ginseng exhibited promising results for improving glucose metabolism^24^. In addition, studies of several ginsenosides in animal models have also showed that these compounds can promote metabolic health and modulate glucose and fat metabolism^54–56^. Taken together, the finding that ginseng metabolite PPD induces Sesn2, AMPK activation, and autophagy provides mechanistic insights into the beneficial effects of ginseng to promote metabolic health.

## Supplementary information

Table S1

Table S2

Fig. S1
