# Supplementary material for: Ginseng metabolite Protopanaxadiol induces Sestrin2 expression and AMPK activation through GCN2 and PERK
Source: Cell Death Dis. 2019 Apr 5;10(4):311. doi: 10.1038/s41419-019-1548-7 (PMC6450862; doi:10.1038/s41419-019-1548-7)
Supplement: Supplementary file 1 — Table S1 [file 41419_2019_1548_MOESM1_ESM.pdf]

Table S1, Major pathways significantly altered by treatment by 20-25 µM of Protopanaxadiol.

	Kegg Pathway	Pathway description	ProbeSet	Name	EntrezID	Parametric p-value	Geom mean of intensities in class 1	Geom mean of intensities in class 2	Fold-change
1	hsa00970	Aminoacyl-tRNA biosynthesis	8104760	threonyl-tRNA synthetase	6897	4.40E-06	1094.96	2091.03	0.52
2		Aminoacyl-tRNA biosynthesis	7914563	tyrosyl-tRNA synthetase	8565	6.00E-06	1014.58	2120.22	0.48
3		Aminoacyl-tRNA biosynthesis	7956443	methionyl-tRNA synthetase	4141	7.50E-06	861.08	1716.2	0.5
4		Aminoacyl-tRNA biosynthesis	8002347	alanyl-tRNA synthetase	16	7.80E-06	1243.34	2683.69	0.46
5		Aminoacyl-tRNA biosynthesis	8132070	glycyl-tRNA synthetase	2617	8.30E-06	3428.43	6017.71	0.57
6		Aminoacyl-tRNA biosynthesis	8114861	leucyl-tRNA synthetase	51520	2.56E-05	1335.66	2098.29	0.64
7		Aminoacyl-tRNA biosynthesis	7903619	seryl-tRNA synthetase	6301	8.82E-05	1495.76	2693	0.56
8		Aminoacyl-tRNA biosynthesis	8023481	asparaginyl-tRNA synthetase	4677	0.000116	3508.57	5537.43	0.63
9		Aminoacyl-tRNA biosynthesis	7981290	tryptophanyl-tRNA synthetase	7453	0.0005524	1468.37	2460.95	0.6
10		Aminoacyl-tRNA biosynthesis	7907466	aspartyl-tRNA synthetase 2, mitochondrial	55157	0.0199142	810.87	598.41	1.36
1	hsa04510	Focal adhesion	8008237	integrin, alpha 3 (antigen CD49C, alpha 3 subunit of VLA-3 receptor)	3675	2.50E-06	496.85	1086.14	0.46
2		Focal adhesion	8119898	vascular endothelial growth factor A	7422	9.60E-06	371.36	926.08	0.4
3		Focal adhesion	8009951	integrin, beta 4	3691	2.64E-05	362.04	586.1	0.62
4		Focal adhesion	8059905	collagen, type VI, alpha 3	1293	3.25E-05	68.91	151.69	0.45

5		Focal adhesion	7909967	calpain 2, (m/II) large subunit	824	9.73E-05	1795.29	2759.13	0.65
6		Focal adhesion	7908072	laminin, gamma 2	3918	0.0001464	284.05	768.02	0.37
7		Focal adhesion	7967002	paxillin	5829	0.0001737	443.67	704.28	0.63
8		Focal adhesion	8128956	FYN oncogene related to SRC, FGR, YES	2534	0.0002553	227.54	438.06	0.52
9		Focal adhesion	8126371	cyclin D3	896	0.0003144	853.16	584.07	1.46
10		Focal adhesion	7978932	son of sevenless homolog 2 (Drosophila)	6655	0.0004649	953.22	1746.2	0.55
11		Focal adhesion	8096301	secreted phosphoprotein 1	6696	0.0006172	175.26	97.34	1.8
12		Focal adhesion	8087337	laminin, beta 2 (laminin S)	3913	0.0008291	213.78	338.97	0.63
13		Focal adhesion	8123584	myosin light chain kinase family, member 4	340156	0.0013858	45.78	72.76	0.63
14		Focal adhesion	8075910	ras-related C3 botulinum toxin substrate 2 (rho family, small GTP binding protein Rac2)	5880	0.0029127	345.69	560.28	0.62
15		Focal adhesion	7982663	budding uninhibited by benzimidazoles 1 homolog beta (yeast)	701	0.004916	2282.92	1093.7	2.09
16		Focal adhesion	7916609	jun proto-oncogene	3725	0.0074261	103.73	197.4	0.53
17		Focal adhesion	7920664	thrombospondin 3	7059	0.0127135	62.97	109.14	0.58
1	hsa04110	Cell cycle	8146357	minichromosome maintenance complex component 4	4173	8.80E-06	699.41	369.65	1.89
2		Cell cycle	7916167	origin recognition complex, subunit 1	4998	1.38E-05	470.05	227.54	2.07
3		Cell cycle	7995631	retinoblastoma-like 2 (p130)	5934	3.97E-05	224.41	380.04	0.59
4		Cell cycle	8151871	cyclin E2	9134	7.04E-05	1722.16	809	2.13

5		Cell cycle	8086880	cell division cycle 25 homolog A (S. pombe)	993	8.36E-05	376.54	198.09	1.9
6		Cell cycle	8082350	minichromosome maintenance complex component 2	4171	9.73E-05	574.7	373.51	1.54
7		Cell cycle	7902227	growth arrest and DNA-damage-inducible, alpha	1647	0.0001232	425.59	840.44	0.51
8		Cell cycle	8160452	cyclin-dependent kinase inhibitor 2B (p15, inhibits CDK4)	1030	0.0001364	392.53	680.29	0.58
9		Cell cycle	8064844	proliferating cell nuclear antigen	5111	0.0001987	897.64	510.23	1.76
10		Cell cycle	8127031	minichromosome maintenance complex component 3	4172	0.0002199	1850.03	1180.35	1.57
11		Cell cycle	8119088	cyclin-dependent kinase inhibitor 1A (p21, Cip1)	1026	0.0002271	190.02	329.7	0.58
12		Cell cycle	7984364	SMAD family member 3	4088	0.0002897	631.8	1120.56	0.56
13		Cell cycle	8126371	cyclin D3	896	0.0003144	853.16	584.07	1.46
14		Cell cycle	8104912	S-phase kinase-associated protein 2 (p45)	6502	0.0003503	3436.36	1833.01	1.87
15		Cell cycle	7998886	protein kinase, membrane associated tyrosine/threonine 1	9088	0.0004565	241.07	152.22	1.58
16		Cell cycle	7995354	origin recognition complex, subunit 6	23594	0.0004595	332.37	182.28	1.82
17		Cell cycle	8072687	minichromosome maintenance complex component 5	4174	0.0005653	783.25	530.06	1.48
18		Cell cycle	8055426	minichromosome maintenance complex component 6	4175	0.0008163	839.47	562.22	1.49
19		Cell cycle	7956076	cyclin-dependent kinase 2	1017	0.0009774	1251.98	840.44	1.49
20		Cell cycle	8066136	retinoblastoma-like 1 (p107)	5933	0.0013906	831.75	508.46	1.64
21		Cell cycle	7938348	WEE1 homolog (S. pombe)	7465	0.0017253	859.09	608.87	1.41

22		Cell cycle	7955736	extra spindle pole bodies homolog 1 (S. cerevisiae)	9700	0.0025593	506.12	234.75	2.16
23		Cell cycle	8102560	MAD2 mitotic arrest deficient-like 1 (yeast)	4085	0.0028623	1562.89	800.63	1.95
24		Cell cycle	8060675	cell division cycle 25 homolog B (S. pombe)	994	0.0028758	578.7	360.79	1.6
25		Cell cycle	7902913	cell division cycle 7 homolog (S. cerevisiae)	8317	0.0030222	339.36	217.52	1.56
26		Cell cycle	8109639	pituitary tumor-transforming 1	9232	0.00565	442.64	200.16	2.21
27		Cell cycle	8024485	growth arrest and DNA-damage-inducible, beta	4616	0.0060352	191.78	309.76	0.62
28		Cell cycle	8054580	budding uninhibited by benzimidazoles 1 homolog (yeast)	699	0.0063957	2586.29	1148.07	2.25
29		Cell cycle	8071212	cell division cycle 45 homolog (S. cerevisiae)	8318	0.0069192	570.73	336.63	1.7
30		Cell cycle	8105828	cyclin B1	891	0.0082631	1382.76	556.41	2.49
31		Cell cycle	7900699	cell division cycle 20 homolog (S. cerevisiae)	991	0.0084226	3947.34	2264.54	1.74
32		Cell cycle	8102643	cyclin A2	890	0.0085168	2139.91	1074.91	1.99
33		Cell cycle	7927710	cyclin-dependent kinase 1	983	0.0087158	1144.1	501.46	2.28
34		Cell cycle	8075164	CHK2 checkpoint homolog (S. pombe)	11200	0.0095036	200.85	136.24	1.47
35		Cell cycle	7994109	polo-like kinase 1	5347	0.0120454	3319.31	1418.35	2.34
36		Cell cycle	8114425	cell division cycle 25 homolog C (S. pombe)	995	0.0141369	225.97	133.9	1.69
37		Cell cycle	7983969	cyclin B2	9133	0.0159237	1675.06	837.53	2
38		Cell cycle	7938364	WEE1 homolog (S. pombe)	7465	0.0335867	1084.89	762.72	1.42

39		Cell cycle	7938366	WEE1 homolog (S. pombe)	7465	0.083817	373.94	279.17	1.34
1	hsa00260	Glycine, serine and threonine metabolism	8156043	phosphoserine aminotransferase 1	29968	2.50E-06	1035.9	2538.92	0.41
2		Glycine, serine and threonine metabolism	8104760	threonyl-tRNA synthetase	6897	4.40E-06	1094.96	2091.03	0.52
3		Glycine, serine and threonine metabolism	8132070	glycyl-tRNA synthetase	2617	8.30E-06	3428.43	6017.71	0.57
4		Glycine, serine and threonine metabolism	8112822	dimethylglycine dehydrogenase	29958	2.20E-05	163.9	398.93	0.41
5		Glycine, serine and threonine metabolism	7904433	phosphoglycerate dehydrogenase	26227	2.32E-05	127.12	236.39	0.54
6		Glycine, serine and threonine metabolism	7903619	seryl-tRNA synthetase	6301	8.82E-05	1495.76	2693	0.56
7		Glycine, serine and threonine metabolism	8013243	serine hydroxymethyltransferase 1 (soluble)	6470	0.0043296	484.38	295.09	1.64
8		Glycine, serine and threonine metabolism	7902290	cystathionase (cystathionine gamma-lyase)	1491	0.0068195	316.63	458.25	0.69
9		Glycine, serine and threonine metabolism	8087419	aminomethyltransferase	275	0.045245	103.25	141.04	0.73
1	hsa00252	Alanine and aspartate metabolism	8141150	asparagine synthetase (glutamine-hydrolyzing)	440	2.00E-07	381.8	1746.2	0.22
2		Alanine and aspartate metabolism	8002347	alanyl-tRNA synthetase	16	7.80E-06	1243.34	2683.69	0.46
3		Alanine and aspartate metabolism	7995362	glutamic pyruvate transaminase (alanine aminotransferase) 2	84706	1.40E-05	138.46	341.32	0.41
4		Alanine and aspartate metabolism	8023481	asparaginyl-tRNA synthetase	4677	0.000116	3508.57	5537.43	0.63
5		Alanine and aspartate metabolism	7907466	aspartyl-tRNA synthetase 2, mitochondrial	55157	0.0199142	810.87	598.41	1.36
1	hsa04810	Regulation of actin cytoskeleton	8008237	integrin, alpha 3 (antigen CD49C, alpha 3 subunit of VLA-3 receptor)	3675	2.50E-06	496.85	1086.14	0.46
2		Regulation of actin cytoskeleton	7950023	fibroblast growth factor 19	9965	6.20E-06	290.69	948.83	0.31

3		Regulation of actin cytoskeleton	8009951	integrin, beta 4	3691	2.64E-05	362.04	586.1	0.62
4		Regulation of actin cytoskeleton	7967002	paxillin	5829	0.0001737	443.67	704.28	0.63
5		Regulation of actin cytoskeleton	7978932	son of sevenless homolog 2 (Drosophila)	6655	0.0004649	953.22	1746.2	0.55
6		Regulation of actin cytoskeleton	8067007	thymosin beta 4, X-linked	7114	0.0009852	677.15	1024	0.66
7		Regulation of actin cytoskeleton	8077612	tubulin tyrosine ligase-like family, member 3	26140	0.0012499	204.6	315.17	0.65
8		Regulation of actin cytoskeleton	8123584	myosin light chain kinase family, member 4	340156	0.0013858	45.78	72.76	0.63
9		Regulation of actin cytoskeleton	8097256	fibroblast growth factor 2 (basic)	2247	0.0017944	498	719.08	0.69
10		Regulation of actin cytoskeleton	8075910	ras-related C3 botulinum toxin substrate 2 (rho family, small GTP binding protein Rac2)	5880	0.0029127	345.69	560.28	0.62
11		Regulation of actin cytoskeleton	7982663	budding uninhibited by benzimidazoles 1 homolog beta (yeast)	701	0.004916	2282.92	1093.7	2.09
12		Regulation of actin cytoskeleton	7971866	diaphanous homolog 3 (Drosophila)	81624	0.0064141	424.61	228.33	1.86
1	hsa04010	MAPK signaling pathway	7964460	DNA-damage-inducible transcript 3	1649	4.80E-06	319.57	1031.12	0.31
2		MAPK signaling pathway	7950023	fibroblast growth factor 19	9965	6.20E-06	290.69	948.83	0.31
3		MAPK signaling pathway	8180337	activating transcription factor 4 (tax-responsive enhancer element B67)	468	1.00E-05	1195.45	2749.59	0.43
4		MAPK signaling pathway	8055992	activin A receptor, type IC	130399	4.72E-05	379.16	214.52	1.77
5		MAPK signaling pathway	7930413	dual specificity phosphatase 5	1847	5.27E-05	877.14	1814.05	0.48
6		MAPK signaling pathway	8032392	MAP kinase interacting serine/threonine kinase 2	2872	0.0001166	463.58	903.89	0.51
7		MAPK signaling pathway	7902227	growth arrest and DNA-damage-inducible, alpha	1647	0.0001232	425.59	840.44	0.51

8		MAPK signaling pathway	7908351	phospholipase A2, group IVA (cytosolic, calcium-dependent)	5321	0.0002001	258.38	120.26	2.15
9		MAPK signaling pathway	7978932	son of sevenless homolog 2 (Drosophila)	6655	0.0004649	953.22	1746.2	0.55
10		MAPK signaling pathway	8097256	fibroblast growth factor 2 (basic)	2247	0.0017944	498	719.08	0.69
11		MAPK signaling pathway	7913869	stathmin 1	3925	0.0025176	2015.14	1278.29	1.58
12		MAPK signaling pathway	8060675	cell division cycle 25 homolog B (S. pombe)	994	0.0028758	578.7	360.79	1.6
13		MAPK signaling pathway	8075910	ras-related C3 botulinum toxin substrate 2 (rho family, small GTP binding protein Rac2)	5880	0.0029127	345.69	560.28	0.62
14		MAPK signaling pathway	7924450	dual specificity phosphatase 10	11221	0.003716	97.01	154.88	0.63
15		MAPK signaling pathway	8024485	growth arrest and DNA-damage-inducible, beta	4616	0.0060352	191.78	309.76	0.62
16		MAPK signaling pathway	8073148	activating transcription factor 4 (tax-responsive enhancer element B67)	468	0.006408	982.29	1573.76	0.62
17		MAPK signaling pathway	7916609	jun proto-oncogene	3725	0.0074261	103.73	197.4	0.53
18		MAPK signaling pathway	8014794	calcium channel, voltage-dependent, beta 1 subunit	782	0.0102328	285.37	398.93	0.72
19		MAPK signaling pathway	7930074	nuclear factor of kappa light polypeptide gene enhancer in B-cells 2 (p49/p100)	4791	0.0241534	667.83	948.83	0.7
20		MAPK signaling pathway	8115831	dual specificity phosphatase 1	1843	0.0778036	458.25	680.29	0.67
21		MAPK signaling pathway	7975779	FBJ murine osteosarcoma viral oncogene homolog	2353	0.0959262	176.48	226.76	0.78
1	hsa00240	Pyrimidine metabolism	8132725	uridine phosphorylase 1	7378	1.72E-05	147.71	524.57	0.28
2		Pyrimidine metabolism	8120967	5'-nucleotidase, ecto (CD73)	4907	1.88E-05	407.31	913.33	0.45
3		Pyrimidine metabolism	7978846	polymerase (DNA directed), epsilon 2 (p59 subunit)	5427	0.0001732	879.17	459.84	1.91

4		Pyrimidine metabolism	7998129	polymerase (RNA) III (DNA directed) polypeptide K, 12.3 kDa	51728	0.0001843	984.56	643.59	1.53
5		Pyrimidine metabolism	7959052	replication factor C (activator 1) 5, 36.5kDa	5985	0.0004101	1005.25	641.36	1.57
6		Pyrimidine metabolism	7949746	polymerase (DNA-directed), delta 4	57804	0.0004706	265.64	429.05	0.62
7		Pyrimidine metabolism	7958174	thioredoxin reductase 1	7296	0.0004804	1548.51	2280.29	0.68
8		Pyrimidine metabolism	7942527	polymerase (DNA-directed), delta 3, accessory subunit	10714	0.000526	488.88	328.56	1.49
9		Pyrimidine metabolism	7937915	ribonucleotide reductase M1	6240	0.0008224	1964.57	1045.52	1.88
10		Pyrimidine metabolism	8095574	deoxycytidine kinase	1633	0.0018572	557.7	295.09	1.89
11		Pyrimidine metabolism	7941214	polymerase (DNA directed), alpha 2 (70kD subunit)	23649	0.0018716	624.55	390.72	1.6
12		Pyrimidine metabolism	8040223	ribonucleotide reductase M2	6241	0.0021029	829.83	377.41	2.2
13		Pyrimidine metabolism	8019842	thymidylate synthetase	7298	0.0021849	2492.42	1101.31	2.26
14		Pyrimidine metabolism	7967736	polymerase (DNA directed), epsilon	5426	0.0028191	642.11	396.18	1.62
15		Pyrimidine metabolism	8018849	thymidine kinase 1, soluble	7083	0.0031611	1424.92	831.75	1.71
16		Pyrimidine metabolism	8166525	polymerase (DNA directed), alpha 1, catalytic subunit	5422	0.0034912	655.6	427.56	1.53
17		Pyrimidine metabolism	8120411	primase, DNA, polypeptide 2 (58kDa)	5558	0.0058811	1272.4	792.35	1.61
18		Pyrimidine metabolism	7964271	primase, DNA, polypeptide 1 (49kDa)	5557	0.0063192	1398.83	879.17	1.59
19		Pyrimidine metabolism	8052331	polyribonucleotide nucleotidyltransferase 1	87178	0.0069741	685.02	489.44	1.4
1	hsa04910	Insulin signaling pathway	7973530	phosphoenolpyruvate carboxykinase 2 (mitochondrial)	5106	8.50E-06	138.46	668.6	0.21

2		Insulin signaling pathway	8145889	eukaryotic translation initiation factor 4E binding protein 1	1978	8.70E-06	309.4	657.11	0.47
3		Insulin signaling pathway	8032392	MAP kinase interacting serine/threonine kinase 2	2872	0.0001166	463.58	903.89	0.51
4		Insulin signaling pathway	8019392	fatty acid synthase	2194	0.0004537	1385.96	784.16	1.77
5		Insulin signaling pathway	7978932	son of sevenless homolog 2 (Drosophila)	6655	0.0004649	953.22	1746.2	0.55
6		Insulin signaling pathway	7938629	phosphodiesterase 3B, cGMP-inhibited	5140	0.0017985	251.31	114.96	2.19
7		Insulin signaling pathway	7992474	tuberous sclerosis 2	7249	0.002979	446.75	636.93	0.7
8		Insulin signaling pathway	7919305	protein kinase, AMP-activated, beta 2 non-catalytic subunit	5565	0.0035244	744.43	1086.14	0.69
1	hsa00230	Purine metabolism	8120967	5'-nucleotidase, ecto (CD73)	4907	1.88E-05	407.31	913.33	0.45
2		Purine metabolism	7978846	polymerase (DNA directed), epsilon 2 (p59 subunit)	5427	0.0001732	879.17	459.84	1.91
3		Purine metabolism	7998129	polymerase (RNA) III (DNA directed) polypeptide K, 12.3 kDa	51728	0.0001843	984.56	643.59	1.53
4		Purine metabolism	8122222	phosphodiesterase 7B	27115	0.0002933	46.42	71.75	0.65
5		Purine metabolism	7959052	replication factor C (activator 1) 5, 36.5kDa	5985	0.0004101	1005.25	641.36	1.57
6		Purine metabolism	7949746	polymerase (DNA-directed), delta 4	57804	0.0004706	265.64	429.05	0.62
7		Purine metabolism	7942527	polymerase (DNA-directed), delta 3, accessory subunit	10714	0.000526	488.88	328.56	1.49
8		Purine metabolism	8088458	fragile histidine triad gene	2272	0.0005777	178.94	320.68	0.56
9		Purine metabolism	7937915	ribonucleotide reductase M1	6240	0.0008224	1964.57	1045.52	1.88
10		Purine metabolism	8122099	ectonucleotide pyrophosphatase/phosphodiesterase 1	5167	0.0011599	2029.16	1327.96	1.53

11		Purine metabolism	8166049	phosphoribosyl pyrophosphate synthetase 2	5634	0.0012414	1284.21	814.63	1.58
12		Purine metabolism	7938629	phosphodiesterase 3B, cGMP-inhibited	5140	0.0017985	251.31	114.96	2.19
13		Purine metabolism	8095574	deoxycytidine kinase	1633	0.0018572	557.7	295.09	1.89
14		Purine metabolism	7941214	polymerase (DNA directed), alpha 2 (70kD subunit)	23649	0.0018716	624.55	390.72	1.6
15		Purine metabolism	8040223	ribonucleotide reductase M2	6241	0.0021029	829.83	377.41	2.2
16		Purine metabolism	7967736	polymerase (DNA directed), epsilon	5426	0.0028191	642.11	396.18	1.62
17		Purine metabolism	8166525	polymerase (DNA directed), alpha 1, catalytic subunit	5422	0.0034912	655.6	427.56	1.53
18		Purine metabolism	8169984	hypoxanthine phosphoribosyltransferase 1	3251	0.0057483	692.98	479.37	1.45
19		Purine metabolism	8120411	primase, DNA, polypeptide 2 (58kDa)	5558	0.0058811	1272.4	792.35	1.61
20		Purine metabolism	7964271	primase, DNA, polypeptide 1 (49kDa)	5557	0.0063192	1398.83	879.17	1.59
21		Purine metabolism	8052331	polyribonucleotide nucleotidyltransferase 1	87178	0.0069741	685.02	489.44	1.4
1	hsa03320	PPAR signaling pathway	7973530	phosphoenolpyruvate carboxykinase 2 (mitochondrial)	5106	8.50E-06	138.46	668.6	0.21
2		PPAR signaling pathway	7930498	acyl-CoA synthetase long-chain family member 5	51703	9.40E-06	113.25	191.34	0.59
3		PPAR signaling pathway	8111941	3-hydroxy-3-methylglutaryl-CoA synthase 1 (soluble)	3157	3.05E-05	861.08	451.94	1.91
4		PPAR signaling pathway	8077899	peroxisome proliferator-activated receptor gamma	5468	0.0005427	294.07	513.78	0.57
5		PPAR signaling pathway	7929816	stearoyl-CoA desaturase (delta-9-desaturase)	6319	0.0010084	5256.91	3565.78	1.47
6		PPAR signaling pathway	7951271	matrix metallopeptidase 1 (interstitial collagenase)	4312	0.0320294	41.84	71.51	0.59

1	hsa00910	Nitrogen metabolism	8141150	asparagine synthetase (glutamine-hydrolyzing)	440	2.00E-07	381.8	1746.2	0.22
2		Nitrogen metabolism	8147132	carbonic anhydrase II	760	1.98E-05	1000.61	541.19	1.85
3		Nitrogen metabolism	8166184	carbonic anhydrase VB, mitochondrial	11238	6.61E-05	277.56	442.64	0.63
4		Nitrogen metabolism	7902290	cystathionase (cystathionine gamma-lyase)	1491	0.0068195	316.63	458.25	0.69
5		Nitrogen metabolism	8087419	aminomethyltransferase	275	0.045245	103.25	141.04	0.73
1	hsa04650	Natural killer cell mediated cytotoxicity	8122724	UL16 binding protein 1	80329	5.40E-06	449.86	978.89	0.46
2		Natural killer cell mediated cytotoxicity	8149733	tumor necrosis factor receptor superfamily, member 10b	8795	3.56E-05	381.8	910.17	0.42
3		Natural killer cell mediated cytotoxicity	7961151	killer cell lectin-like receptor subfamily K, member 1	22914	0.0002416	42.42	91.14	0.47
4		Natural killer cell mediated cytotoxicity	8128956	FYN oncogene related to SRC, FGR, YES	2534	0.0002553	227.54	438.06	0.52
5		Natural killer cell mediated cytotoxicity	7978932	son of sevenless homolog 2 (Drosophila)	6655	0.0004649	953.22	1746.2	0.55
6		Natural killer cell mediated cytotoxicity	8075910	ras-related C3 botulinum toxin substrate 2 (rho family, small GTP binding protein Rac2)	5880	0.0029127	345.69	560.28	0.62
7		Natural killer cell mediated cytotoxicity	7961173	KLRC4-KLRK1 readthrough	1E+08	0.0086581	51.27	75.85	0.68
1	hsa00620	Pyruvate metabolism	7973530	phosphoenolpyruvate carboxykinase 2 (mitochondrial)	5106	8.50E-06	138.46	668.6	0.21
2		Pyruvate metabolism	7958784	aldehyde dehydrogenase 2 family (mitochondrial)	217	4.51E-05	606.07	1332.57	0.45
3		Pyruvate metabolism	8062041	acyl-CoA synthetase short-chain family member 2	55902	0.0002361	405.44	731.64	0.55
4		Pyruvate metabolism	8155327	aldehyde dehydrogenase 1 family, member B1	219	0.0002392	1348.06	795.1	1.7
5		Pyruvate metabolism	8123137	acetyl-CoA acetyltransferase 2	39	0.0002444	857.11	266.87	3.21

6		Pyruvate metabolism	8113773	aldehyde dehydrogenase 7 family, member A1	501	0.024013	1026.37	736.73	1.39
1	hsa04350	TGF-beta signaling pathway	7995631	retinoblastoma-like 2 (p130)	5934	3.97E-05	224.41	380.04	0.59
2		TGF-beta signaling pathway	8055992	activin A receptor, type IC	130399	4.72E-05	379.16	214.52	1.77
3		TGF-beta signaling pathway	8160452	cyclin-dependent kinase inhibitor 2B (p15, inhibits CDK4)	1030	0.0001364	392.53	680.29	0.58
4		TGF-beta signaling pathway	7979241	bone morphogenetic protein 4	652	0.000142	2784.75	1552.09	1.79
5		TGF-beta signaling pathway	7984364	SMAD family member 3	4088	0.0002897	631.8	1120.56	0.56
6		TGF-beta signaling pathway	8066136	retinoblastoma-like 1 (p107)	5933	0.0013906	831.75	508.46	1.64
7		TGF-beta signaling pathway	7920664	thrombospondin 3	7059	0.0127135	62.97	109.14	0.58
1	hsa04360	Axon guidance	7932985	neuropilin 1	8829	1.90E-06	205.07	962.07	0.21
2		Axon guidance	7956759	SLIT-ROBO Rho GTPase activating protein 1	57522	6.70E-06	709.18	1418.35	0.5
3		Axon guidance	8140668	sema domain, immunoglobulin domain (Ig), short basic domain, secreted, (semaphorin) 3A	10371	0.0002462	98.82	55.14	1.79
4		Axon guidance	8128956	FYN oncogene related to SRC, FGR, YES	2534	0.0002553	227.54	438.06	0.52
5		Axon guidance	8109093	actin binding LIM protein family, member 3	22885	0.0007353	265.03	413	0.64
6		Axon guidance	8075910	ras-related C3 botulinum toxin substrate 2 (rho family, small GTP binding protein Rac2)	5880	0.0029127	345.69	560.28	0.62
7		Axon guidance	8040725	dihydropyrimidinase-like 5	56896	0.0036374	226.49	158.13	1.43
8		Axon guidance	7982663	budding uninhibited by benzimidazoles 1 homolog beta (yeast)	701	0.004916	2282.92	1093.7	2.09
9		Axon guidance	8170921	plexin A3	55558	0.0339977	280.14	420.22	0.67

1	hsa04512	ECM-receptor interaction	8008237	integrin, alpha 3 (antigen CD49C, alpha 3 subunit of VLA-3 receptor)	3675	2.50E-06	496.85	1086.14	0.46
2		ECM-receptor interaction	8009951	integrin, beta 4	3691	2.64E-05	362.04	586.1	0.62
3		ECM-receptor interaction	8059905	collagen, type VI, alpha 3	1293	3.25E-05	68.91	151.69	0.45
4		ECM-receptor interaction	7908072	laminin, gamma 2	3918	0.0001464	284.05	768.02	0.37
5		ECM-receptor interaction	7939341	CD44 molecule (Indian blood group)	960	0.0004451	940.1	1646.29	0.57
6		ECM-receptor interaction	8096301	secreted phosphoprotein 1	6696	0.0006172	175.26	97.34	1.8
7		ECM-receptor interaction	8087337	laminin, beta 2 (laminin S)	3913	0.0008291	213.78	338.97	0.63
8		ECM-receptor interaction	7920664	thrombospondin 3	7059	0.0127135	62.97	109.14	0.58
9		ECM-receptor interaction	8109712	hyaluronan-mediated motility receptor (RHAMM)	3161	0.016962	444.69	238.86	1.86
10		ECM-receptor interaction	8071272	SEPT5-GP1BB readthrough	1E+08	0.4148921	114.04	129.79	0.88
1	hsa00640	Propanoate metabolism	7958784	aldehyde dehydrogenase 2 family (mitochondrial)	217	4.51E-05	606.07	1332.57	0.45
2		Propanoate metabolism	7916432	24-dehydrocholesterol reductase	1718	5.16E-05	3708.62	2120.22	1.75
3		Propanoate metabolism	8062041	acyl-CoA synthetase short-chain family member 2	55902	0.0002361	405.44	731.64	0.55
4		Propanoate metabolism	8155327	aldehyde dehydrogenase 1 family, member B1	219	0.0002392	1348.06	795.1	1.7
5		Propanoate metabolism	8123137	acetyl-CoA acetyltransferase 2	39	0.0002444	857.11	266.87	3.21
6		Propanoate metabolism	8113773	aldehyde dehydrogenase 7 family, member A1	501	0.024013	1026.37	736.73	1.39
1	hsa01430	Cell Communication	8009951	integrin, beta 4	3691	2.64E-05	362.04	586.1	0.62

2		Cell Communication	8059905	collagen, type VI, alpha 3	1293	3.25E-05	68.91	151.69	0.45
3		Cell Communication	7908072	laminin, gamma 2	3918	0.0001464	284.05	768.02	0.37
4		Cell Communication	8154725	keratin 18	3875	0.0003613	1502.69	2418.67	0.62
5		Cell Communication	8096301	secreted phosphoprotein 1	6696	0.0006172	175.26	97.34	1.8
6		Cell Communication	8087337	laminin, beta 2 (laminin S)	3913	0.0008291	213.78	338.97	0.63
7		Cell Communication	8107706	lamin B1	4001	0.0015245	508.46	218.27	2.33
8		Cell Communication	8015349	keratin 19	3880	0.0021687	637.67	1049.15	0.61
9		Cell Communication	7920664	thrombospondin 3	7059	0.0127135	62.97	109.14	0.58
10		Cell Communication	8032491	lamin B2	84823	0.0130967	1382.76	972.13	1.42
1	hsa04310	Wnt signaling pathway	8135763	wingless-type MMTV integration site family, member 16	51384	1.90E-06	399.86	192.67	2.08
2		Wnt signaling pathway	7951614	protein phosphatase 2, regulatory subunit A, beta	5519	0.0002786	1178.99	765.36	1.54
3		Wnt signaling pathway	7984364	SMAD family member 3	4088	0.0002897	631.8	1120.56	0.56
4		Wnt signaling pathway	8126371	cyclin D3	896	0.0003144	853.16	584.07	1.46
5		Wnt signaling pathway	8102415	calcium/calmodulin-dependent protein kinase II delta	817	0.0007214	670.92	1031.12	0.65
6		Wnt signaling pathway	8060897	phospholipase C, beta 4	5332	0.0012531	175.66	299.21	0.59
7		Wnt signaling pathway	8076734	wingless-type MMTV integration site family, member 7B	7477	0.0020281	108.63	166	0.65
8		Wnt signaling pathway	8075910	ras-related C3 botulinum toxin substrate 2 (rho family, small GTP binding protein Rac2)	5880	0.0029127	345.69	560.28	0.62

9		Wnt signaling pathway	8167287	porcupine homolog (Drosophila)	64840	0.0041132	333.91	531.9	0.63
10		Wnt signaling pathway	7927631	dickkopf homolog 1 (Xenopus laevis)	22943	0.0049623	6280.51	3942.79	1.59
11		Wnt signaling pathway	7916609	jun proto-oncogene	3725	0.0074261	103.73	197.4	0.53
1	hsa00280	Valine, leucine and isoleucine degradation	8111941	3-hydroxy-3-methylglutaryl-CoA synthase 1 (soluble)	3157	3.05E-05	861.08	451.94	1.91
2		Valine, leucine and isoleucine degradation	7958784	aldehyde dehydrogenase 2 family (mitochondrial)	217	4.51E-05	606.07	1332.57	0.45
3		Valine, leucine and isoleucine degradation	8155327	aldehyde dehydrogenase 1 family, member B1	219	0.0002392	1348.06	795.1	1.7
4		Valine, leucine and isoleucine degradation	8123137	acetyl-CoA acetyltransferase 2	39	0.0002444	857.11	266.87	3.21
5		Valine, leucine and isoleucine degradation	8029065	branched chain keto acid dehydrogenase E1, alpha polypeptide	593	0.0008492	562.87	922.88	0.61
6		Valine, leucine and isoleucine degradation	8162264	AU RNA binding protein/enoyl-CoA hydratase	549	0.0015864	444.69	639.15	0.7
7		Valine, leucine and isoleucine degradation	8145570	establishment of cohesion 1 homolog 2 (S. cerevisiae)	157570	0.0028265	2515.57	1433.18	1.76
8		Valine, leucine and isoleucine degradation	8113773	aldehyde dehydrogenase 7 family, member A1	501	0.024013	1026.37	736.73	1.39
1	hsa04520	Adherens junction	8055992	activin A receptor, type IC	130399	4.72E-05	379.16	214.52	1.77
2		Adherens junction	8019988	protein tyrosine phosphatase, receptor type, M	5797	0.0002105	81.95	154.88	0.53
3		Adherens junction	8128956	FYN oncogene related to SRC, FGR, YES	2534	0.0002553	227.54	438.06	0.52
4		Adherens junction	7984364	SMAD family member 3	4088	0.0002897	631.8	1120.56	0.56
5		Adherens junction	8150698	snail homolog 2 (Drosophila)	6591	0.0008562	717.42	1112.82	0.64
6		Adherens junction	8075910	ras-related C3 botulinum toxin substrate 2 (rho family, small GTP binding protein Rac2)	5880	0.0029127	345.69	560.28	0.62

1	hsa00100	Biosynthesis of steroids	8106280	3-hydroxy-3-methylglutaryl-CoA reductase	3156	8.36E-05	1640.59	945.54	1.74
2		Biosynthesis of steroids	7931754	isopentenyl-diphosphate delta isomerase 1	3422	0.0001611	1816.15	1009.9	1.8
3		Biosynthesis of steroids	7905986	farnesyl diphosphate synthase	2224	0.0003583	401.71	243.03	1.65
4		Biosynthesis of steroids	8167305	emopamil binding protein (sterol isomerase)	10682	0.0005033	948.83	594.28	1.6
5		Biosynthesis of steroids	7950067	7-dehydrocholesterol reductase	1717	0.000942	1344.95	823.14	1.63
1	hsa00650	Butanoate metabolism	8111941	3-hydroxy-3-methylglutaryl-CoA synthase 1 (soluble)	3157	3.05E-05	861.08	451.94	1.91
2		Butanoate metabolism	7958784	aldehyde dehydrogenase 2 family (mitochondrial)	217	4.51E-05	606.07	1332.57	0.45
3		Butanoate metabolism	8155327	aldehyde dehydrogenase 1 family, member B1	219	0.0002392	1348.06	795.1	1.7
4		Butanoate metabolism	8123137	acetyl-CoA acetyltransferase 2	39	0.0002444	857.11	266.87	3.21
5		Butanoate metabolism	8083415	arylacetamide deacetylase (esterase)	13	0.0020909	80.45	237.21	0.34
6		Butanoate metabolism	8113773	aldehyde dehydrogenase 7 family, member A1	501	0.024013	1026.37	736.73	1.39
1	hsa04540	Gap junction	8101284	protein kinase, cGMP-dependent, type II	5593	4.25E-05	60.83	122.79	0.5
2		Gap junction	8165496	tubulin, beta 2C	10383	0.0001322	119.7	64.67	1.85
3		Gap junction	7978932	son of sevenless homolog 2 (Drosophila)	6655	0.0004649	953.22	1746.2	0.55
4		Gap junction	7989657	casein kinase 1, gamma 1	53944	0.0004859	359.54	196.04	1.83
5		Gap junction	8059177	tubulin, alpha 4a	7277	0.0005943	891.44	610.99	1.46
6		Gap junction	8060897	phospholipase C, beta 4	5332	0.0012531	175.66	299.21	0.59

7		Gap junction	7955179	tubulin, alpha 1c	84790	0.0043307	3640.7	2610.3	1.39
8		Gap junction	7927710	cyclin-dependent kinase 1	983	0.0087158	1144.1	501.46	2.28
1	hsa04020	Calcium signaling pathway	8063942	neurotensin receptor 1 (high affinity)	4923	4.99E-05	588.13	1176.27	0.5
2		Calcium signaling pathway	7929388	phospholipase C, epsilon 1	51196	5.61E-05	380.92	226.76	1.68
3		Calcium signaling pathway	8102415	calcium/calmodulin-dependent protein kinase II delta	817	0.0007214	670.92	1031.12	0.65
4		Calcium signaling pathway	8060897	phospholipase C, beta 4	5332	0.0012531	175.66	299.21	0.59
5		Calcium signaling pathway	8123584	myosin light chain kinase family, member 4	340156	0.0013858	45.78	72.76	0.63
6		Calcium signaling pathway	8097692	endothelin receptor type A	1909	0.0016368	176.07	298.17	0.59
7		Calcium signaling pathway	8107307	calcium/calmodulin-dependent protein kinase IV	814	0.0020472	646.57	380.04	1.7
8		Calcium signaling pathway	8083166	transient receptor potential cation channel, subfamily C, member 1	7220	0.0030796	223.89	337.79	0.66
1	hsa00380	Tryptophan metabolism	7958784	aldehyde dehydrogenase 2 family (mitochondrial)	217	4.51E-05	606.07	1332.57	0.45
2		Tryptophan metabolism	7916432	24-dehydrocholesterol reductase	1718	5.16E-05	3708.62	2120.22	1.75
3		Tryptophan metabolism	8155327	aldehyde dehydrogenase 1 family, member B1	219	0.0002392	1348.06	795.1	1.7
4		Tryptophan metabolism	8123137	acetyl-CoA acetyltransferase 2	39	0.0002444	857.11	266.87	3.21
5		Tryptophan metabolism	7981290	tryptophanyl-tRNA synthetase	7453	0.0005524	1468.37	2460.95	0.6
6		Tryptophan metabolism	8024900	ubiquitin-like with PHD and ring finger domains 1	29128	0.0007114	1123.15	670.92	1.67
7		Tryptophan metabolism	8080001	HemK methyltransferase family member 1	51409	0.0045964	151.87	243.03	0.62

8		Tryptophan metabolism	8035095	cytochrome P450, family 4, subfamily F, polypeptide 11	57834	0.0134138	94.57	200.85	0.47
9		Tryptophan metabolism	8113773	aldehyde dehydrogenase 7 family, member A1	501	0.024013	1026.37	736.73	1.39
1	hsa04912	GnRH signaling pathway	8180337	activating transcription factor 4 (tax-responsive enhancer element B67)	468	1.00E-05	1195.45	2749.59	0.43
2		GnRH signaling pathway	7908351	phospholipase A2, group IVA (cytosolic, calcium-dependent)	5321	0.0002001	258.38	120.26	2.15
3		GnRH signaling pathway	7978932	son of sevenless homolog 2 (Drosophila)	6655	0.0004649	953.22	1746.2	0.55
4		GnRH signaling pathway	8102415	calcium/calmodulin-dependent protein kinase II delta	817	0.0007214	670.92	1031.12	0.65
5		GnRH signaling pathway	8060897	phospholipase C, beta 4	5332	0.0012531	175.66	299.21	0.59
6		GnRH signaling pathway	8073148	activating transcription factor 4 (tax-responsive enhancer element B67)	468	0.006408	982.29	1573.76	0.62
7		GnRH signaling pathway	7916609	jun proto-oncogene	3725	0.0074261	103.73	197.4	0.53
1	hsa03030	DNA polymerase	7978846	polymerase (DNA directed), epsilon 2 (p59 subunit)	5427	0.0001732	879.17	459.84	1.91
2		DNA polymerase	7959052	replication factor C (activator 1) 5, 36.5kDa	5985	0.0004101	1005.25	641.36	1.57
3		DNA polymerase	7949746	polymerase (DNA-directed), delta 4	57804	0.0004706	265.64	429.05	0.62
4		DNA polymerase	7942527	polymerase (DNA-directed), delta 3, accessory subunit	10714	0.000526	488.88	328.56	1.49
5		DNA polymerase	7941214	polymerase (DNA directed), alpha 2 (70kD subunit)	23649	0.0018716	624.55	390.72	1.6
6		DNA polymerase	7967736	polymerase (DNA directed), epsilon	5426	0.0028191	642.11	396.18	1.62
7		DNA polymerase	8021275	polymerase (DNA directed) iota	11201	0.0030394	959.85	1384.36	0.69
8		DNA polymerase	8166525	polymerase (DNA directed), alpha 1, catalytic subunit	5422	0.0034912	655.6	427.56	1.53

9		DNA polymerase	8120411	primase, DNA, polypeptide 2 (58kDa)	5558	0.0058811	1272.4	792.35	1.61
10		DNA polymerase	7964271	primase, DNA, polypeptide 1 (49kDa)	5557	0.0063192	1398.83	879.17	1.59
11		DNA polymerase	8089875	polymerase (DNA directed), theta	10721	0.0080501	343.3	184.82	1.86
1	hsa00564	Glycerophospholipid metabolism	7908351	phospholipase A2, group IVA (cytosolic, calcium-dependent)	5321	0.0002001	258.38	120.26	2.15
2		Glycerophospholipid metabolism	7956046	diacylglycerol kinase, alpha 80kDa	1606	0.0014659	760.08	1184.45	0.64
3		Glycerophospholipid metabolism	8145570	establishment of cohesion 1 homolog 2 (S. cerevisiae)	157570	0.0028265	2515.57	1433.18	1.76
4		Glycerophospholipid metabolism	8045816	glycerol-3-phosphate dehydrogenase 2 (mitochondrial)	2820	0.0028422	1344.95	873.1	1.54
5		Glycerophospholipid metabolism	8130628	1-acylglycerol-3-phosphate O-acyltransferase 4 (lysophosphatidic acid acyltransferase, delta)	56895	0.003299	146.36	254.23	0.58
