# Supplementary material for: Ginseng metabolite Protopanaxadiol induces Sestrin2 expression and AMPK activation through GCN2 and PERK
Source: Cell Death Dis. 2019 Apr 5;10(4):311. doi: 10.1038/s41419-019-1548-7 (PMC6450862; doi:10.1038/s41419-019-1548-7)
Supplement: Supplementary file 2 — Table S2 [file 41419_2019_1548_MOESM2_ESM.pdf]

Table S2, List of genes identified by microarray significance analysis with a false discovery rate of 0.001

Geom mean of intensities in class 1	Geom mean of intensities in class 2	Fold-change	ProbeSet	Symbol	Name
697.8	4640.29	0.15	8102800	SLC7A11	solute carrier family 7, (cationic amino acid transporter, y+ system) member 11
113.77	781.44	0.15	7982868	CHAC1	ChaC, cation transport regulator homolog 1 (E. coli)
140.72	786.88	0.18	7928308	DDIT4	DNA-damage-inducible transcript 4
116.43	726.59	0.16	7958262	TCP11L2	t-complex 11 (mouse)-like 2
381.8	1746.2	0.22	8141150	ASNS	asparagine synthetase (glutamine-hydrolyzing)
79.71	414.43	0.19	8154381	C9orf150	chromosome 9 open reading frame 150
578.7	2926.58	0.2	8006531	SLFN5	schlafen family member 5
205.07	962.07	0.21	7932985	NRP1	neuropilin 1
138.46	668.6	0.21	7973530	PCK2	phosphoenolpyruvate carboxykinase 2 (mitochondrial)
166.96	675.59	0.25	8115851	STC2	stanniocalcin 2
28.44	132.06	0.22	7954398	C12orf39	chromosome 12 open reading frame 39
118.06	541.19	0.22	7899436	SESN2	sestrin 2
37.27	132.06	0.28	8082058	CSTA	cystatin A (stefin A)
326.29	1152.06	0.28	8143629	OR2A9P	olfactory receptor, family 2, subfamily A, member 9 pseudogene
334.69	1184.45	0.28	8136983	OR2A20P	olfactory receptor, family 2, subfamily A, member 20 pseudogene
178.94	726.59	0.25	8008454	ABCC3	ATP-binding cassette, sub-family C (CFTR/MRP), member 3
115.09	394.81	0.29	7915543	SLC6A9	solute carrier family 6 (neurotransmitter transporter, glycine), member 9
147.71	524.57	0.28	8132725	UPP1	uridine phosphorylase 1
146.36	528.22	0.28	7922846	FAM129A	family with sequence similarity 129, member A
290.69	948.83	0.31	7950023	FGF19	fibroblast growth factor 19
319.57	1031.12	0.31	7964460	DDIT3	DNA-damage-inducible transcript 3

674.03	2344.4	0.29	7965423	BTG1	B-cell translocation gene 1, anti-proliferative
131.6	427.57	0.31	8016476	HOXB9	homeobox B9
324.03	1002.93	0.32	8020955	MOCOS	molybdenum cofactor sulfurase
483.26	1370.04	0.35	8060353	RBCK1	RanBP-type and C3HC4-type zinc finger containing 1
108.89	405.91	0.27	8135069	SERPINE1	serpin peptidase inhibitor, clade E (nexin, plasminogen activator inhibitor type 1), member 1
193.56	517.35	0.37	7945803	NA	NA
261.38	795.1	0.33	8027002	GDF15	growth differentiation factor 15
85.83	237.21	0.36	8020971	NA	NA
310.83	846.29	0.37	8063386	CEBPB	CCAAT/enhancer binding protein (C/EBP), beta
281.44	770.69	0.37	8170119	FHL1	four and a half LIM domains 1
