# Supplementary material for: Ginseng metabolite Protopanaxadiol induces Sestrin2 expression and AMPK activation through GCN2 and PERK
Source: Cell Death Dis. 2019 Apr 5;10(4):311. doi: 10.1038/s41419-019-1548-7 (PMC6450862; doi:10.1038/s41419-019-1548-7)
Supplement: Supplementary file 3 — Fig. S1 [file 41419_2019_1548_MOESM3_ESM.pdf]

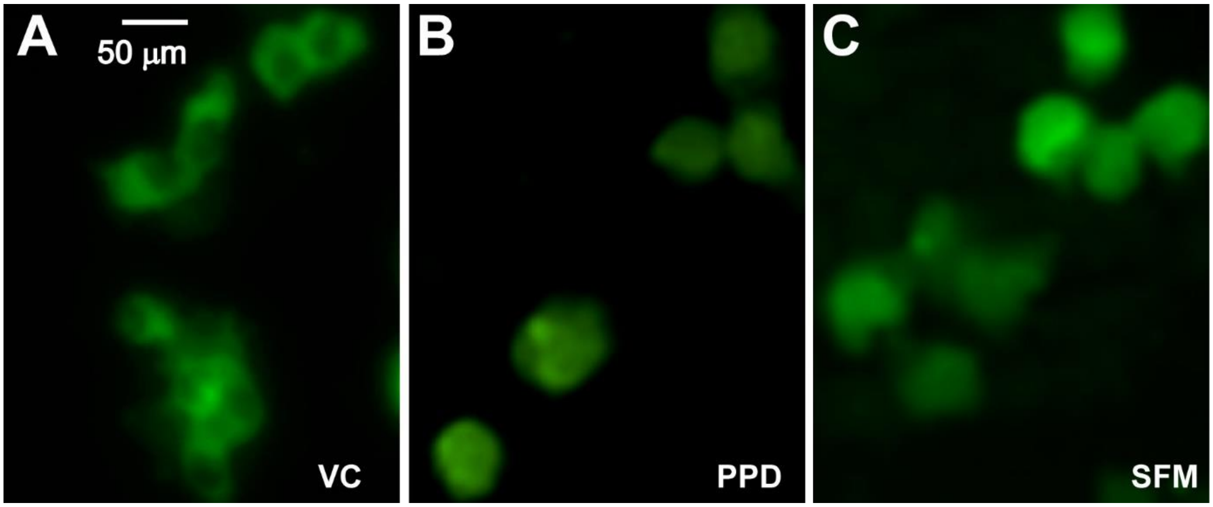

Figure S1. PPD and Serum deprivation treatment induce TFEB nuclear translocation. HCT116 cells were transfected with TFEB-GFP plasmid. 48 hours after transfection, cells were treated with vehicle control (VC), PPD 30 µM (PPD) or Serum-free medium (SFM) for 20 h, and then the localization of TFEB-GFP were analyzed by fluorescence microscopy. TFEB-GFP was excluded from the nucleus in vehicle control treated cells but was present in the nucleus as well as in the cytoplasm in either PPD or serum free medium treated cells. White scale bar indicate 50 µm.
